# Chronic Lymphocytic Leukaemia in 2020: the Future Has Arrived

**DOI:** 10.1007/s11912-020-0893-0

**Published:** 2020-03-14

**Authors:** Kate Milne, Beattie Sturrock, Timothy Chevassut

**Affiliations:** 1 Brighton and Sussex Medical School, University of Sussex, Brighton, BN1 9PS UK; 2 Royal Sussex County Hospital, Eastern Road, Brighton, BN2 5BE UK

**Keywords:** Chronic lymphocytic leukaemia, Chemoimmunotherapy, Ibrutinib, Venetoclax, Future

## Abstract

**Purpose of Review:**

Chronic lymphocytic leukaemia is now recognised as a heterogenous disease with a variety of clinical outcomes. Here we summarise the way it is currently stratified according to genetic risk and patient characteristics and the treatment approaches used for these different subgroups.

**Recent Findings:**

Certain patients appear to sustain MRD negativity after combination chemoimmunotherapy, leading to the suggestion that their CLL may be cured. However, 17p-deleted, p53-mutated or IGHV-UM subgroups are generally resistant to FCR, and much better responses are seen with ibrutinib and venetoclax, frequently inducing MRD negativity that hopefully will be translated into durable remissions.

**Summary:**

Small molecule inhibitors have already revolutionised CLL treatment. Going forward, we anticipate their use in the majority of patients, early after diagnosis and with curative intent.

## Introduction

Chronic lymphocytic leukaemia (CLL) is the commonest leukaemia in the world, with 4.9 new diagnosis per 100,000 per year in the UK and USA. The malignant clonal proliferation and accumulation of mature B-lymphocytes is predominantly identified in older patients with a median age of 74 at diagnosis [1, 2]. The majority of patients are monitored with a ‘watch and wait approach’ until the balance of risks and benefits favours treatment initiation. In some cases, treatment may never be needed whilst in others, the disease is more aggressive with rapid progression and death from disease-related causes a few years after diagnosis [3]. This disparity in outcome highlights the heterogeneity of CLL and the importance of risk stratification to guide treatment decisions. unlike, its myeloid counterpart—chronic myeloid leukaemia—a pathognomonic driving mutation, BCR-ABL, has not been identified, and this slightly delayed the development of targeted therapies. However, over the last two decades, a dramatic increase in our understanding of the pathogenesis of the disease has led to the development of small molecule inhibitors for CLL targeting the B cell receptor pathway and the apoptotic regulator BCL2. Some of these newer therapies appear to be so effective; they may provide a curative option for patients, previously treatment aimed to establish disease control. In this review, we will briefly discuss recent advances in our understanding of the molecular pathology of CLL and then, in more detail, the way that CLL is managed in the UK.

## New Developments in the Understanding of the Pathogenies of CLL

Our understanding of the genetics of CLL and the implications on patient outcome began with the publication by Doner et al. in 2000 which identified four recurrent genetic lesions by fluorescence in situ hybridisation (FISH) and showing that their presence predicted disease prognosis. It was found that a lesion at 17p13 and 11q23 indicated a poor prognosis compared with 13q14 and 12q; this formed the basis of the FISH probe set used routinely during CLL diagnostics [4]. The utility of FISH is limited by the need to identify particular region to probe and by the number of probes that can be used at one time. Unlike FISH, G-banding karyotyping which can be applied to all the chromosomes simultaneously, required cells to be in metaphase, various compounds have been used to raise the yield of metaphase cells in CLL—the combination of DSp30/IL2 has shown to do so without inducing artefactual chromosomal aberrations [5, 6]. Using this technique, it has been demonstrated 16–19% of patients have a complex karyotype (3 > abnormalities) that this predicts poor outcome independent of P53 status, and the presence of 5 of more is associated with a P53 mutations and perhaps due to this association, a more aggressive course [7]. These techniques have been replaced in research by chromosomal micro arrays and single nucleotide polymorphisms arrays which do not require the cells to be in the cell cycle and whilst the concordance of these approaches is reported in small studies, their incorporation into large trials is awaited [8].

CLL patients can be categorised into two groups depending on the mutational status, established by PCR or next generation sequencing, of the variable region immunoglobulin heavy chain (IGHV) when compared with germline sequence. During the process of VDJ rearrangement and somatic hypermutation of B cells occurs within the germinal centres generating receptors capable of recognising an extensive range of antigens. IGHV-mutated (IGHV-M) CLL, with a mutation status of > 2%, compared with germline sequence as an immunophenotype and gene expression profile similar to that of post GC CD27+, T cell dependent memory B cells. Whilst IGHV-unmutated (IGHV-UM) CLL cells adopted a phenotype gene expression profile and epigenome that resemble a GC-naïve CD27− B cell [9, 10]. This difference is reinforced when comparing the BCRs of the two subtypes with IGHV-UM carrying low affinity, poly-reactive and self-reactive BCR, and IGHV-M has higher affinity, olio or mono reactive receptors [11]. This may contribute to the poorer prognosis seen with IGHV-UM CLL [12, 13].

Next generation sequencing confirmed that the mutational rate in CLL is similar to that of other haematological cancers and lower than solid malignancy, with each patients having a small number of recurrently mutated driver genes—unsurprisingly, many of the mutated genes identified in these studies are involved in cell replication, DNA repair apoptosis and signalling [14–17]. The subtypes, the 4 chromosomal abnormalities discussed earlier have also been shown to be associated with different recurrent gene mutations, similarly to IGHV status, which may explain the heterogeneity in there clinical course. For example, 17p13 deletion occurs with mutation of the remaining p53 resulting in homozygous inactivity and SF3B1, a protein involved in the regulation of the spliceosome with 11q deletion [18, 19]. In these studies, a quarter of patients had a mutation in a gene involved in RNA splicing or repair—a possible avenue for new drug development [20].

As well as identification of mutated genes in CLL, the mechanisms of gene expression regulation have also been studied. It is known that BCL2 an antiapoptotic protein is overexpressed in CLL and that its expression increases with chemotherapy [21–23]. BCL2 combines with BH3 and binds to BIM, preventing BIM triggering apoptosis [24]. The recurrent deletion of 13q14 is seen in 50% of de novo CLL; it has been shown that this deletion results in the loss of expression of micro RNA (miRNA) 15-A/16-1 [25, 26]. As miRNA 15-A and 16-1 normal interfere in the transcription of BCL2, their loss contributes to its overexpression in CLL [27].

Interaction with other cells in the microenvironment is crucial to the survival and replication of CLL cells, as shown by the rapid apoptosis of CLL cells in vitro and the reduction in this when co-cultured with non-tumoral bystander cells [11]. The lymph nodes can be the key site of CLL proliferation, with higher activation of the NFKb and the BCR signalling pathways crucial for this process than the bone marrow of peripheral blood [28], whilst the bone marrow creates a protective niches preventing spontaneous and drug-induced apoptosis cells [29, 30]. Interruption of these interactions may force the cells into the peripheral circulation, therefore increasing their susceptibility to drug-induced chemotherapy.

## Treatment of Chronic Lymphocytic Leukaemia in 2019

Most patients with CLL present with an incidental finding of a lymphocytosis, they may have palpable lymphadenopathy or organomegaly, bone marrow involvement can lead to anaemia and thrombocytopenia. Occasionally, patients may have the constitutional B symptoms with an unexplained fever of over 38°, weight loss of > 10% in less than 6 months and night sweats. A complication of CLL such as a high-grade disease transformation, an autoimmune disease or a severe infection may lead to its diagnosis [31].

The following diagnostic criteria are stipulated by the international workshop of CLL (iwCLL) and reiterated in the WHO classification of lymphoid neoplasms. The lymphocytes count in the peripheral blood must be greater than 5 × 10 × 9, persistent for 3 months, and clonality of this population must be shown by flow cytometry for light chain restriction, CD5, CD23, CD79b and surface immunoglobulin expression with low levels of CD20. The presence of smudge/smear cells, an artefact of blood film production, is a typical finding in CLL [32]. If the WCC is less than 5 × 10/L with no other signs of lymphoproliferative disorder, this constitutes monoclonal B cell lymphocytosis which is thought as a precursor to CLL, with a rate of progression of 1% per year [33]. Identification of a clonal population of mature B cells within the lymph nodes or extranodal tissues without a peripheral blood lymphocytosis is referred to as a small lymphocytic lymphoma [34]. Fluorescence in situ hybridisation is used to identify chromosomal rearrangements which can differentiate CLL from other conditions such as mantle cell lymphoma as well as helping with disease stratification.

## When to Initiate Treatment

In addition to diagnostic criteria, the iwCLL guidelines dictate when to initiate treatment, based on patients’ symptoms, full blood counts and physical examination. The presence of constitutional symptoms, progressive lymphocytosis, a doubling time of less than 6 months, an Hb of less than 100 g/L or a platelet count of less than 100 x 10^9^/L as well as progressive or symptomatic bulky lymphadenopathy/organomegaly or treatment-resistant autoimmune thrombocytopaenia or anaemia are indications to start treatment [32]. In some circumstances, treatment may be initiated at diagnosis, but it is much more common for patient to be monitored for signs of increasing disease activity often over many years.

## Patient Risk Stratification

**Fig. 1:**
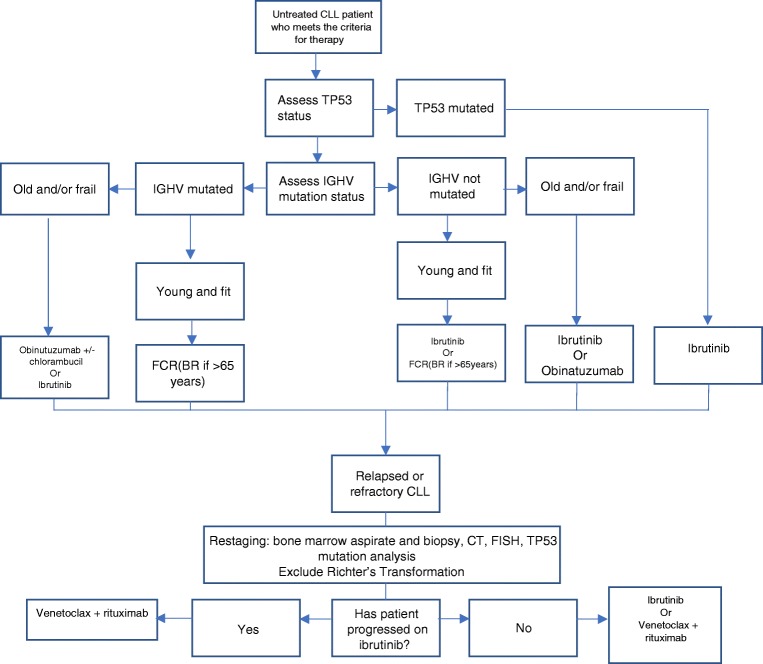
Flow chart showing the decision process and treatment options in management of previously untreated CLL that meets the iwCLL criteria for treatment and in relapsed or refractory disease

Similar to the management of most malignancy, the most suitable treatment for a patient with CLL is selected based on genetic features of the disease itself—the presence of a P53 mutation or 17p deletion and the mutational state of the IGHV—and patient factors [35]. The algorithm for treatment selection followed by haematologists in the UK is shown in Fig. 1. Disruption of P53, a tumour suppressor crucial in many cancers, is known to result in very poor response to combination immunochemotherapy, and therefore, patients are treated with molecular therapies first line [18]. If the IGHV is unmutated, compared with the germline sequence, this confers a poor survival risk when compared with patient with a mutated IGHV [12, 13]. The co-morbidities and performance status of individual patients are crucial in setting treatment goals and treatment selection. Treatment success or disease progression is determined on similar criteria to treatment initiation—repeat CT scans are not recommended out with clinical trials. The identification of minimal residual disease (MRD) on peripheral blood or bone marrow aspirate by flow cytometry, with a sensitivity of 0.01%, is becoming increasingly important as MRD negativity after chemoimmunotherapy is associated with prolonged progression-free and overall survival—in the future, early evidence of MRD negativity may allow for shorter treatment regimens [36, 37].

Upon treatment failure on combination chemoimmunotherapy or ibrutinib, there are now several available options for relapsed disease including the PI3K inhibitor idelalisib and the BCL2 inhibitor Venetoclax, also shown in Fig. 1.

## Combination Immunochemotherapy—Fludarabine, Cyclophosphamide, Rituximab (FCR) and Other CD20 Antibodies

Young fit patients, with a creatine clearance of greater than 70 and a comorbidity scale of 6 or less without P53 mutations and with mutated IGHV, are treated with combination chemoimmunotherapy, fludarabine, cyclophosphamide and rituximab (FCR) [38, 39]. Prior to the introduction of immunotherapy chlorambucil (CLB), an alkylating agent or fludarabine, a purine analogue monotherapy was the mainstay of treatment—the combination of these agents did not improve response rates but did carry much higher haematological toxicity [40]. The addition of cyclophosphamide to fludarabine (FC) led to an improved progression-free survival (PFS) and overall survival (OS) without an increase in serious adverse events compared with fludarabine monotherapy [41]. The approval of rituximab—a monoclonal antibody targeting CD20, a glycosylated cell surface protein expressed on mature B cells—provided a treatment option for many B cell malignancies. In the CLL8 trial, patients were randomised to receive either FC or FCR, which gave a response rate of 85 and 92% respectively. Particular subgroups of patients appear to have particularly good long-term outcomes, those with a mutated IGVH, del(13q), trisomy 12 or del(11q) or MRD negative remission [42, 43•]. The durability of these remissions led to the suggestion that FCR may be curative for some patients. There is emerging evidence that reduced doses or 3 rather than 6 cycles result in non-inferior PFS and OS with a lower burden of cumulative toxicity [36].

Whilst FCR is known to result in longer PFS compared with bendamustine and rituximab (BR) in younger patients, the benefit was not seen in patients over 65—and a lower rate of serious infections were seen in the BR cohort. Therefore, in patients over 65 who do not qualify for ibrutinib BR tended to be favoured, unless there is a contraindication to bendamustine when chlorambucil rituximab could be used accepting that this had a poorer response rate [44, 45]. The CLL11 trial looked specifically at the treatment of older patients with co-morbidities and the impact of a new anti CD20 monoclonal antibody, obinutuzumab. Treatment with obinutuzumab-CLB compared with R-CLB and CLB monthotherapy increased response rates and prolonged PFS (median PFS, 26.7 months with obinutuzumab-CLB vs 11.1 months with CLB alone; 16.3 months with R-CLB) [46]. Currently, results of a large clinical trial multicentre trial, with over 900 participants are awaited, comparing single-agent obinutuzumab and its combination with FC, chlorambucil or bendamustine in untreated and relapsed CLL is awaited; it is possible that obinutuzumab will replace rituximab in the long-standing FCR regime [47, 48].

## Bruton Tyrosine Kinase Inhibitors—Ibrutinib

The B cell receptor and downstream signalling pathways are crucial to the survival and proliferation of malignant cells in CLL. Unlike their healthy counterparts in CLL, signalling from the B cell receptor is activated independently of appropriate antigen stimulation—autologous activation. Bruton tyrosine kinase (BTK) which is only slightly downstream of the receptor itself activates the cell survival pathway NfK-B and MAP kinases, its inhibition leads to apoptosis of CLL cells [49]. Ibrutinib is an orally available small molecular inhibitor that binds to BTK preventing its kinase activity. This affects multiple signalling pathways and disrupts the interactions between CLL cells and the microenvironment leading to further apoptosis.

Initially trialled in relapsed or refractory CLL, response rates to ibrutinib were between 84 and 97% and complete response rates were 12–23% [50]. This was independent of previously identified poor prognostic factors including advanced stage disease, number of previous lines of treatment and del 17(p). The RESONATE trial, a multicentre phase 3 open-label trial with 391 participants, compared ibrutinib with an anti-CD20 antibody, ofatumumab, note no longer in use, in a cohort of patients with relapsed CLL/SLL. Ibrutinib significantly improved the response rate, PFS and OS. The median PFS of 8.1 months in the ofatumumab group whilst at 9.4 months, the median PFS had not been reached in the ibrutinib group, PFS of 88% at 6 months [51]. RESONATE 2 showed that ibrutinib was more effective than single-agent chlorambucil as a first-line treatment in patients aged over 65. Ibrutinib significantly prolonged overall survival; estimated survival rate at 24 months was 98% with ibrutinib and 85% with chlorambucil, with a relative risk of death that was 84% lower in the ibrutinib group. The overall response rate was higher with ibrutinib than with chlorambucil (86% vs 35%) [51, 52]. It is important to note that unlike RESONATE in RESONATE 2, the presence of a 17p deletion was an exclusion criterion, and the use of single-agent chlorambucil without a CD20 antibody is now very rare. A more relevant comparison of ibrutinib versus bendamustine and rituximab in patients over 65 did show ibrutinib to confer a significant benefit. The estimated percentage of patients with progression-free survival at 2 years was 74% with bendamustine plus rituximab and 87% with ibrutinib alone. Combined rituximab and ibrutinib provided no additional benefit compared with ibrutinib alone with an estimated PFS of 88% at 2 years [53]. Ibrutinib and obinutuzumab does appear to be beneficial compared with CLB-obinutuzumab with a high estimated 30-month overall survival and fewer serious adverse events in the iLLUMINATE phase 3 trial of first-line treatment [54] (Table 1).

**Table 1:** Significant randomised and more recent phase 1/2 trials using targeted small molecular inhibitors in CLL

Treatment	FL/RR	Number	Age	ORR	NMRD	2YOS	Reference
Randomised phase 3 trials							
Ibrutinib	RR	195	67	63%	NR	1 year 90%	Byrd 2014 [51]
Ofatumumab		196	67	4%	NR	1 year 81%	
Ibrutinib	FL	136	73	86%	NR	98%	Burger 2015 [52]
Chorambucil		133	72	35%	NR	85%	
Ibrutinib	FL	182	71	93%	1%	90%	Woyach 2018 [53]
Ibrutinib/rituximab		182	71	94%	4%	94%	
Ibrutinib/obinutuzumab	FL	113	70	88%	35%	30 m 86%	Moreno 2019 [54]
CLB/obinutuzumab		116	72	73%	25%	30 m 85%	
Bendamustine/rituximab		183	70	81%	8%	95%	
Ibrutinib/rituximab	FL	354	58	96%	8%	3 years 99%	Shanfelt 2019 [55]
FCR		175	57	81%	59%	3 years 92%	
Idelalisib/rituximab	RR	110	71	81%	NR	1 year 92%	Furman 2014 [62]
Rituximab		110	71	13%	NR	1 year 80%	
Venetoclax/rituximab	RR	194	65	92%	62%	92%	Seymour 2018 [68]
Bendamustine/rituximab		195	65	72%	13%	87%	
Phase 1/2 trials							
Ibrutinib/venetoclax	FL	80	65	88%	61%	1 year 99%	Jain 2019 [73]
Alcalabrutinib	RR	61	62	95%	NR	1 year 100%	Byrd 2016 [75]

*FL* first-line therapy, *RR* relapsed or refractory, *Age* median in years, *ORR* overall response rate, *NMRD* negative MRD in peripheral blood, *2****YOS*** 2-year overall survival rate unless alternate follow-up period specified

The use of ibrutinib first line in younger patients, without 17p deletion or p53 mutation, has not yet been clearly demonstrated, one trial has reported an improvement in PFS and OS with ibrutinib and rituximab compared with FCR as a first-line treatment of CLL in patients under 70 [55]. However, as of yet, there has only been a short follow-up period, and there was a surprisingly high number of deaths in the FCR arm indicating further work is needed.

The results of the RESONATE trials have not been replicated in the clinic; the UK ibrutinib real world study reported that 44% of the patients had a dose reduction, interruption of complete cessation in the first 12 months compared with 4% in the resonate study. The OS at 12 months was 83%, 89% for patients with no dose reduction or cessation of less than 14 days compared with 90% in the RESONATE [56•]. A theoretical benefit of small molecule inhibitors is the reduced side effect profile but due to off-target effects, these are still not negligible with significant bleeding, recurrent infections and cardiac toxicity, particular atrial fibrillation, being the most common reasons for treatment interruption or cessation.

We are now beginning to understand the mechanisms underlying the ibrutinib failure to ibrutinib failure. Comparisons of targeted deep sequencing before initiation of ibrutinib and at the point or either CLL progression or Richter’s transformation identified new mutations in BTK or PLG2 that were not present prior to treatment [57]. A larger prospective study also identified these mutations in some patients who had not yet shown signs of clinical relapse suggesting sequencing may become an indicator of when further intervention is required [58].

## Phosphatidylinositol 3-Kinases(PI3K) Inhibitors—Idelalisib

The PI3K signalling pathway, downstream of the B cell receptor, is constitutively activated in CLL and is required for their survival and proliferation [59, 60]. Idelalisib is a potent and specific inhibitor of PI3K isoform expression of which is restricted to cells of haematopoietic origin. Idelalisib induces apoptosis in CLL cells whilst T cells and NK cell are unaffected. Like ibrutinib, idelalisib has multiple mechanisms of action, such as disruption of the CLL cell CXC12 and CXC13 driven chemotaxis towards stromal cells and their migration beneath them; this may keep the cells within the peripheral blood increasing their susceptibility to apoptosis induction [60]. Idelalisib was initially evaluated in relapsed and refractory disease including patients with adverse features—bulky lymphadenopathy, 17p deletion/Tp53 mutation, IGHV-unmutated and failure of multiple treatments. Idelalisib had an overall response rate of 72% in this cohort and PFS of 15.8 months [61]. The combination of idelalisib with rituximab, compared with rituximab alone, leads to higher overall response rate 81%vs 13% and a 12-month survival or 92% vs 81%. There was also a higher rate of reported serious adverse events in the idelalisib and rituximab group (40%)—the most common being pneumonia, pyrexia and febrile neutropoenia; it is likely that due to its toxicity, its use will be restricted to relapsed disease [62].

## BCL2 Inhibition—Venetoclax

An ability to evade apoptosis is required for the development of cancer—making its regulatory pathways an important therapeutic target [63]. Venetoclax, a BH3 mimic, prevents the interaction between BCL2 and BH3 inducing cell death [64]. Earlier BH3 mimetics showed good disease response but induced severe thrombocytopaenia in a phase 1 trial [65]. Venetoclax avoids this due to its higher specificity for BCL2 than some of its predecessors. The phase 1 and 2 trials of venetoclax showed impressive results with an overall response rate of 70–80% across all prognostic groups including patients with a 17p deletion/Tp53 mutation. Additionally, unlike the use of ibrutinib and idelalisib, venetoclax-induced MRD negative complete responses [66]. The most important adverse effect was the occurrence of fatal tumour lysis syndrome in the initial phase 1 trial; this could occur after even a single dose of 100 or 200 mg. Since the introduction of a strict dosing increment regime, there have been no further deaths attributable to, and a lower incidence of TLS. Monitoring for cytopaenias, infection and hepatotoxicity is also required.

A retrospective analysis of the UK patients started on venetoclax, who had failed a BTK inhibitor and/or a PI3K inhibitor, reported an overall response rate of 88%. At the median follow-up of 15·6 months, the 1-year PFS and OS was 65.0% and 75.1%, respectively. Of particular interest was the response rate of 80% in patients who had received both BTK and PI3K inhibitors—a group of patients who previously had no further treatment options other than autologous stem cell transplant [67]. A similar study conducted in the US reported a lower ORR of 72%, but a much higher proportion of patients therapy was held/stopped 29% compared with 8% in the UK, and so far, the follow-up period has been significantly shorter—7 months [3]. The use of venetoclax in combination with monthly rituximab had a dramatically improved outcome at 2 years compared with BR in relapsed CLL, and this combination is now commonly used in clinical practice [68••]. Interestingly, the VR regimes result in 62% of patients having MRD negativity in peripheral blood, compared with 13% of the BR, hopefully longer follow-up will show that this combination results in a durable remission. Venetoclax is not immune to the development of resistance with identified mechanisms being mutation of BCL2 or the compensatory over expression of MCL1, another BCL family member [69, 70]. However with the promising MRD negativity seen in VR regimes, it is hoped that combination therapy that includes venetoclax may prevent the development of resistance and treatment failure.

As the apoptotic pathways are targeted by venetoclax are universal, there is hope its success in CLL will be replicated. Within haematology, there have been promising results supporting the use of venetoclax in combination with hypomethylating agents such as daunorubicin in relapsed AML or with cytidine analogues as a first-line treatment for AML in older patients [71].

## Conclusion—the Future of CLL Treatment

The last decade has seen dramatic change in CLL treatment but there are important trial results awaited in 2020 and further into the future. The watch and wait principle of CLL—delaying treatment initiation until a progression means the iwCLL criteria are met is from the chemoimmunotherapy era. Several trials challenging this with the initiation of ibrutinib at diagnosis in patients without an indication to treat but high-risk disease are active, with results due to be reported in mid-2020 or in ongoing recruitment [72, 73]. Additionally, longer term follow-up in patients treated with small molecule inhibitors is required to see if the achievement of MRD negativity improves survival as seen with FCR—it is likely that monitoring MRD will become more common place in CLL, as it is already in other haematological malignancies [74]. The results of a multicentre phase 1/2 trials establishing the maximum tolerated dose of alcalabrutinib—a BTK inhibitor with higher specificity and lower reversibility than ibrutinib—are due to be published in early 2021 indicating that with time, we will likely see an increase in the number of available drugs within the classes of small molecule inhibitors already established [75]. Perhaps, the most exciting current trials in CLL are the use of multiple molecular inhibitors—such as venetoclax and ibrutinib simultaneous—this combination has been shown to provoke a complete remission, including MRD negativity, in patients after a limited duration of treatment meaning that patients may not be committed to indefinite therapy [73••]. Limiting length of treatment regimens has the advantageous of reduced exposure, and therefore, toxicity particularly relevant in CLL due to the increasingly complex co-morbidities seen in this patient cohort. It is recommend that fit patients with relapsed CLL, or a 17p deletion, should be considered for allogeneic transplantation after the failure of one kinase inhibitor—whilst venetoclax provides a valid option for these patients in the future CAR T cells may also prevent the need for transplant [76]. Small studies of anti-CD19 CAR T cells in patients who relapsed on BTK inhibitors have shown response rates of over 70% and a survival rate of 100%, although only after 6 months of follow-up. Further larger trials are required, but we can be cautiously optimistic; this may provide a safer alternative to transplantation in patients who fail small molecule therapy [77, 78].
